# Impact of the m.13513G>A Variant on the Functions of the OXPHOS System and Cell Retrograde Signaling

**DOI:** 10.3390/cimb45030115

**Published:** 2023-02-22

**Authors:** Dita Kidere, Pawel Zayakin, Diana Livcane, Marina Makrecka-Kuka, Janis Stavusis, Baiba Lace, Tsu-Kung Lin, Chia-Wei Liou, Inna Inashkina

**Affiliations:** 1 Latvian Biomedical Research and Study Centre, LV-1067 Riga, Latvia; 2 Latvian Institute of Organic Synthesis, LV-1006 Riga, Latvia; 3 Children’s Clinical University Hospital, LV-1004 Riga, Latvia; 4 Department of Neurology, Kaohsiung Chang Gung Memorial Hospital and Chang Gung University College of Medicine, Kaohsiung 83305, Taiwan; 5 Center for Mitochondrial Research and Medicine, Kaohsiung Chang Gung Memorial Hospital and Chang Gung University College of Medicine, Kaohsiung 83301, Taiwan

**Keywords:** OXPHOS system, retrograde signaling, RNA sequencing, mitochondrial diseases, Leigh syndrome

## Abstract

Mitochondria are involved in many vital functions in living cells, including the synthesis of ATP by oxidative phosphorylation (OXPHOS) and regulation of nuclear gene expression through retrograde signaling. Leigh syndrome is a heterogeneous neurological disorder resulting from an isolated complex I deficiency that causes damage to mitochondrial energy production. The pathogenic mitochondrial DNA (mtDNA) variant m.13513G>A has been associated with Leigh syndrome. The present study investigated the effects of this mtDNA variant on the OXPHOS system and cell retrograde signaling. Transmitochondrial cytoplasmic hybrid (cybrid) cell lines harboring 50% and 70% of the m.13513G>A variant were generated and tested along with wild-type (WT) cells. The functionality of the OXPHOS system was evaluated by spectrophotometric assessment of enzyme activity and high-resolution respirometry. Nuclear gene expression was investigated by RNA sequencing and droplet digital PCR. Increasing levels of heteroplasmy were associated with reduced OXPHOS system complex I, IV, and I + III activities, and high-resolution respirometry also showed a complex I defect. Profound changes in transcription levels of nuclear genes were observed in the cell lines harboring the pathogenic mtDNA variant, indicating the physiological processes associated with defective mitochondria.

## 1. Introduction

Mitochondria are dynamic cell structures that possess their own intrinsic complement of circular DNA (mtDNA), ribosomes, and proteins [1]. Mitochondria play essential roles in eukaryotic aerobic metabolism, including in ATP synthesis and fatty acid oxidation, as well as being involved in the biosynthesis of intermediary metabolites, the regulation of calcium homeostasis, citric acid cycle metabolism, heat generation, and apoptosis [2,3,4,5,6,7,8,9]. Mitochondria are also involved in retrograde signaling, a communication system in which mitochondria generate signals that regulate nuclear gene expression [10]. The retrograde response may be stimulated by the ATP/ADT ratio, disruption of the mitochondrial membrane potential, reactive oxygen species (ROS) production, or general cellular stress [11,12]. Mitochondrial dysfunction in mammalian cells results in reductions in mitochondrial membrane potential, incrementally altering intracellular calcium levels [10]. These alterations result in the activation of calcium-dependent kinases and phosphatases (e.g., calcineurin, PKC, CamKIV, JNK, and MAPK), culminating in the activation of various transcription factors, such as ATF2, CEBP/δ, NFAT, CREB, Egr-1, CHOP, and NFκB [10,13,14,15,16].

Mutations in human mtDNA have been associated with various multisystem disorders known as mitochondrial diseases, with the severity of these disorders depending on the mutant mtDNA load in cells [17]. Furthermore, pathogenic variants in mtDNA, reduced mtDNA copy number, or disruption of mitochondrial respiratory chain (RC) complexes activate mitochondria-to-nucleus retrograde signaling, which induces a global change in nuclear gene expression, ultimately contributing to various pathologic conditions. These mitochondrial changes cause transcriptional reprogramming of nuclear genes at the epigenetic [18], transcriptional [19,20,21], and post-transcriptional [22,23] levels, although the exact mechanism and components of this cross-talk remain unclear.

Transmitochondrial cytoplasmic hybrid systems (cybrids) are created by fusing mitochondria-enriched platelets with mitochondria-depleted cells [24,25]. These cybrids allow the expression of selected mtDNA sequences against a fixed nuclear DNA (nDNA) background, providing a method for studying the effects of known mtDNA variants on mitochondrial biochemistry and mtDNA-nDNA crosstalk. RNA seq has been used to evaluate the transcriptional profiles of cybrids with different levels of heteroplasmy of the *MT-TL1* m.3243A>G variant, showing that quantitative changes in mtDNA mutant levels can qualitatively regulate nuclear gene expression [26].

Isolated complex I deficiency represents the most common mitochondrial respiratory chain defect, involved in mitochondrial disorders, and it has been associated with heterogeneous manifestations, including phenotypic overlaps of mitochondrial encephalomyopathy with lactic acidosis and stroke-like episodes (MELAS), Leigh syndrome, and Leber’s hereditary optic neuropathy (LHON) [27]. A previous report from our laboratory described a patient with Leigh syndrome harboring the mtDNA variant m.13513G>A in the *MT-ND5* gene at 60% heteroplasmy level [28]. The present study was designed to investigate the effect of this m.13513G>A variant on the oxidative phosphorylation (OXPHOS) system and cell retrograde signaling. Cybrid cell models differing in heteroplasmy levels were generated, and mitochondrial OXPHOS enzyme activities, oxygen consumption, and transcriptional profiles were analyzed in these cybrid cell lines differing in mutant mtDNA load in comparison to cells harboring the wild-type (WT) variant.

## 2. Materials and Methods

### 2.1. Recruitment of Participants and Ethics Statement

A patient with mitochondrial disease and healthy volunteers were recruited for this study. The study protocols were approved by the Central Committee of Medical Ethics of Latvia (protocol No.2016-5, chapter 2, approved 24 November 2016, and protocol No.2020-6 II, chapter-3, approved 22 May 2020), which covers all consent- and data-handling-related issues for genetic research on human subjects. All participants and/or their legal guardians provided written informed consent prior to enrollment. The study conformed to the guidelines of the Declaration of Helsinki.

### 2.2. Cell Culture, Generation of ρ0 Cells, and Construction of Cybrids

Human 143B osteosarcoma cells were cultured in Dulbecco’s modified Eagle’s medium (DMEM, high glucose, Gibco, Carlsbad, CA, USA) supplemented with 10% heat-inactivated fetal bovine serum (FBS; Gibco) and antibiotic–antimycotic solution (Gibco) at 37 °C in an atmosphere containing 5% CO_2_. Human 143B ρ0 cells were generated by long-term culture of 143B cells with 50 ng/mL ethidium bromide (Merck KGaA, Darmstadt, Germany), 1 mM pyruvate (Gibco), and 50 μg/mL uridine (Sigma, St. Louis, MO, USA) [29]. The cells were subcultured every 2–3 days and kept at 50–70% confluence. The absence of mtDNA was determined by real-time PCR [30].

Transmitochondrial cybrids were generated by fusing 143B ρ0 cells with human platelets in the absence of pyruvate and uridine, as previously reported [24] and modified [25]. Platelets containing the heteroplasmic m.13513G>A variant were isolated and fused with ρ0 cells in the presence of polyethylene glycol 1500 (50% *w*/*v*; Sigma). The mixture of fused cells was allowed to recover for 1 week in ρ0 medium and cultured in pyruvate/uridine-free DMEM, a medium, in which even cybrids with very low Cox-II expression had been shown to grow. On days 14–30 after fusion, successful cybrid clones growing in this medium were isolated. The presence of exogenous mtDNA or specific mtDNA haplogroups in each cybrid was confirmed by Sanger sequencing of isolated DNA [28].

### 2.3. Mitochondria Isolation

Mitochondria were isolated from cytoplasmic hybrid cells as previously described [31], with modifications. Briefly, cells were suspended in 10 mM Tris (pH 7.4) and homogenized with a glass–glass Dounce homogenizer on ice. Sucrose was added immediately to a concentration of 0.25 M, and the suspension was centrifuged at 600× *g* for 10 min at 4 °C. The postnuclear supernatant was decanted and again centrifuged at 10,000× *g* for 10 min at 4 °C. Mitochondria-enriched pellets were washed with a buffer containing 250 mM sucrose, 2 mM EGTA, 40 mM KCl, and 20 mM Tris (pH 7.4); suspended in the same buffer; and stored at −70 °C.

### 2.4. Enzyme Measurements

Enzymatic activities of citrate synthase (CS), OXPHOS complexes I–IV, and complexes I + III and II + III in isolated mitochondria were measured spectrophotometrically (Uvicon 922, Kontron, Italy) at 37 °C, as previously described [32,33,34], with modifications. Briefly, CS activity was measured by adding 10 μL of isolated mitochondria suspension to a reaction mixture containing 50 mM Tris/HCl (pH 8.1), 0.1% bovine serum albumin (BSA), 0.1% TritonX-100, 0.2 mM 5,5′-dithio-bis(2-nitrobenzoic acid) (DTNB), and 0.15 mM acetyl-CoA. Thiolase activity was recorded for 2–3 min, 0.5 mM oxaloacetate was added, and CS activity was recorded for 8 min at 412 nm.

To measure complex I activity, 40 µL of isolated mitochondria were first disrupted by resuspension in water and incubated for 3 min at 37 °C. Then, a reaction mixture containing 50 mM Tris (pH 8.1), 0.1% BSA, 0.3 mM KCN, and 50 µM decylubiquinone was added, and the reaction was initiated by adding 0.2 mM NADH. The NADH oxidation reaction was allowed to proceed for 10 min, and complex I activity was measured at 340 nm, after which 3 µM rotenone, a complex I inhibitor, was added, and nonspecific NADH oxidation was recorded.

To measure complex II activity, 10 µL of isolated mitochondria was added to a reaction mixture containing 50 mM potassium phosphate buffer (pH 7.8), 2 mM ethylenediaminetetraacetic acid (EDTA), 0.1% BSA, 3 µM rotenone, 80 µM 2,6-dichlorophenolindophenol, 50 µM decylubiquinone, 1 µM antimycin A, 0.2 mM ATP, and 0.3 mM KCN. After preincubation for 10 min, the reaction was started by adding 10 mM succinate and allowed to proceed for 5 min, during which complex II activity was measured at 600 nm. Subsequently, 10 mM malonate was added to stop the reaction and determine nonspecific activity.

Complex III activity was measured by adding 5 µL of isolated mitochondria to a reaction mixture containing 50 mM potassium phosphate buffer (pH 7.8), 0.08% Tween-20, 2 mM EDTA, 0.3 mM KCN, 100 µM cytochrome c, and 200 µM reduced decylubiquinol. Cytochrome c reduction was measured at 550 nm for 4 min, after which 1 µM antimycin A was added to inhibit the reaction and determine the nonspecific activity.

The activity of complexes I + III was measured by incubating 10 µL of isolated mitochondria in water for 3 min at 37 °C, followed by addition of a reaction mixture containing 50 mM Tris (pH 8.1), 0.1% BSA, 0.3 mM KCN, and 50 µM cytochrome c. The reaction was started by adding 0.2 mM NADH and allowed to proceed for 3–4 min, with activity measured at 550 nm. The reaction was stopped by adding 3 µM rotenone, and nonspecific activity was determined.

The activity of complexes II + III was determined by incubating 10 µL of isolated mitochondria with a reaction mixture containing 50 mM potassium phosphate buffer (pH 7.8), 2 mM EDTA, 0.1% BSA, 3 µM rotenone, 0.2 mM ATP, and 0.3 mM KCN at 37 °C. The reactions were started by adding 50 µM cytochrome c and 10 mM succinate. Activity was measured for 3 min, and 10 mM malonate was added to stop the reaction and determine nonspecific activity.

Complex IV activity was measured as described [35], with modifications. The reaction was started by adding 60 µM reduced cytochrome c to a reaction mixture containing 50 mM potassium phosphate buffer (pH 7.0) and 0.1% BSA, and 10 µL of isolated mitochondria. Activity was measured at 550 nm for 10 min. Subsequently, 1 µL of bovine heart mitochondria was added to reach the endpoint of the reaction, and the final absorbance at 550 nm was recorded after 30 min.

### 2.5. High-Resolution Respirometry

Mitochondrial functionality was determined by high-resolution respirometry using an OxygraphO2k (O2k; Oroboros Instruments, Innsbruck, Austria), according to the manufacturer’s instructions. All measurements were performed in Mir05 media (110 mM sucrose, 60 mM K-lactobionate, 0.5 mM EGTA, 3 mM MgCl_2_, 20 mM taurine, 10 mM KH_2_PO_4_, 20 mM HEPES, and 0.1% BSA essentially free of fatty acids) at 37 °C. Mitochondrial respiration was initially measured in intact cells (ROUTINE state), followed by the addition of 5 µg/mL digitonin to permeabilize the cells. Complex I-linked oxygen consumption was measured using 5 mM pyruvate plus 2 mM malate (LEAK state), followed by subsequent additions of 5 mM ADP (OXPHOS state) and 10 mM glutamate. Complex II-linked mitochondrial respiration was evaluated by adding 10 mM succinate and 0.5 µM rotenone. Residual oxygen consumption (ROX) was determined by adding 2.5 µM antimycin A, and complex IV-linked oxygen consumption was measured by adding 0.5 mM N,N,N′,N′-tetramethyl-*p*-phenylenediamine dihydrochloride (TMPD) and 2 mM ascorbate. Complex IV activity was inhibited, and chemical background determined by the addition of 100 mM sodium azide. The contribution of each substrate to the respiration rate was determined by calculating the flux control factor using the formula: Flux control factor = 1 − (respiratory rate before the addition of substrate)/(respiratory rate after the addition of substrate).

### 2.6. RNA Sample Preparation and Next Generation Sequencing

Cultivated cybrid cells containing 50% and 70% heteroplasmy of the m.13513G>A variant and cells containing WT m.13513G were collected and washed with PBS. Total RNA was isolated using RNeasy Mini Kits (QIAGEN Science, Germantown, MD, USA), according to the manufacturer’s instructions. The integrity of the extracted RNA was evaluated by determining the RNA integrity number (RIN) using the Agilent 2100 Bioanalyzer system (Agilent, Santa Clara, CA, USA). Ribosomal RNA was removed from 500 ng aliquots of total RNA from each sample using the Low Input RiboMinus Eukaryote System v2 (Thermo Fisher Scientific, Waltham, MA, USA). cDNA libraries were prepared using the Ion Total RNA-Seq Kits v2 (Thermo Fisher Scientific, USA) and sequenced using the Ion Proton System and Ion PI Chip (Thermo Fisher Scientific, USA), according to the manufacturer’s instructions. All experiments were performed on two biological replicates.

### 2.7. Bioinformatic Analysis

The adapters were trimmed from sequencing reads with Cutadapt [36]. For inclusion in subsequent analyses, sequencing reads after trimming were required to be of a minimum length of 42 bp, with reads corresponding to rRNA removed with Sortmerna [37]. Sequencing reads were mapped against the human reference genome GRCh38.p12 with STAR 2.7.1a [38], and read counts were calculated with HTSEQ-count [39]. The obtained read counts were normalized, principal component analysis was performed by package PCAtools [40], and differentially expressed genes (DEGs) were estimated using the Bioconductor package DESeq2 [41] and EnhancedVolcano [42] in R. Multiple testing correction was implemented using the Benjamini–Hochberg procedure, with significant DEGs defined as those having an absolute log fold change (FC) > 2 and a false discovery rate (FDR) < 0.05. GO and enrichment analysis were performed with R packages GOstat [43], rentrez [44], GO.db [45], and ShinyGO [46].

### 2.8. Selfie-Digital RT-PCR

Cell culture media were aspirated from the culture flasks, and each cell layer was lysed in 100ST DNA/RNA/Protein Solubilization Reagent (DireCtQuant, Lleida, Spain) at a concentration of 250,000 cells/mL, to preserve the nucleic acids in the samples. The lysates were incubated at 90 °C for 3 min with shaking and centrifuged at 5000× *g* for 1 min, and the supernatants were used directly. Strand-specific absolute levels of gene transcription were analyzed using Selfie-digital PCR, as described previously [47]. The reverse transcription step was performed using the reverse primer listed in Table S1 and Maxima H Minus Reverse Transcriptase (Thermo Fisher Scientific, USA). Digital PCR was performed using EvaGreen SuperMix (Bio-Rad, Hercules, CA, USA) and the QX200 ddPCR platform. The number of RNA transcripts was calculated by subtracting the number of amplicons measured in the reaction without reverse transcriptase (RT−) from the number measured in the reaction with reverse transcriptase (RT+) and dividing by (RT-). The results are expressed as the number of transcripts per gene. These experiments were repeated twice, with each containing two technical replicates.

## 3. Results

The effects of the genetic variant m.13513G>A in the *MT-ND5* gene on the OXPHOS system and nuclear gene expression in cells were investigated by constructing cybrid cell lines. Platelets from a patient harboring 60% of mutant mtDNA in blood were fused with mtDNA-free ρ0 cells. Heteroplasmic cybrid cell lines containing 50% and 70% of mutant mtDNA were generated, along with a cybrid cell line harboring only wild-type (WT) mtDNA.

### 3.1. Functionality of the OXPHOS System

**Figure 1:**
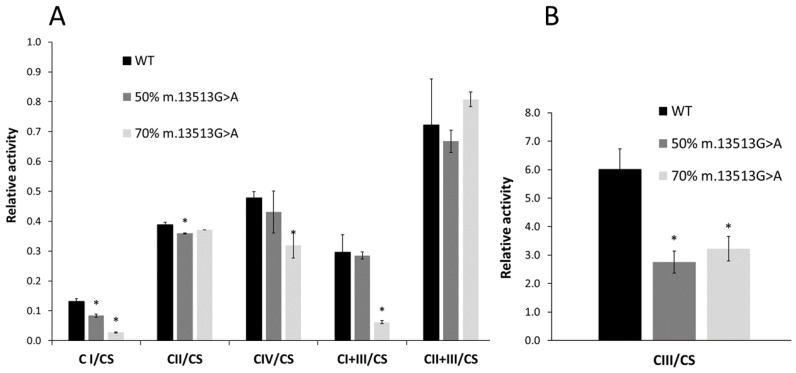
Ratios of OXPHOS system complex activities to citrate synthase (CS) activity in cell lines harboring 50% and 70% of the m.13513G>A variant compared with wild-type (WT) cells. (**A**) Relative activities of the OXPHOS system complexes I, II, IV, I + III, and II + III. (**B**) Relative activity of the OXPHOS system complex III. Data are shown as the mean ± standard deviation. * *p* < 0.05 compared with WT by two-sample t-tests.

To determine whether different heteroplasmy levels of the m.13513G>A variant impaired the performance of the OXPHOS system and mitochondrial respiration, enzyme activities were measured, and high-resolution respirometry was performed. All mitochondrial OXPHOS complex activities were expressed relative to CS activity, as CS is a marker of mitochondrial content [48], as a ratio called “complex activity” throughout the text. Measurements of the activity of enzymes in the OXPHOS system revealed that increasing levels of heteroplasmy caused dose-dependent reductions in complex I activity, with cell lines containing 50% and 70% mutated m.13513G>A showing 38% and 77% reductions, respectively, in complex I activity compared with WT cells (Figure 1A). Similarly, these mutant cell lines showed 54% and 47% reductions, respectively, in complex III activity (Figure 1B). Furthermore, the cell line harboring 70% heteroplasmy showed 67% and 20% reductions in complex IV and complexes I + III activities relative to WT (Figure 1A). All these reductions in OXPHOS complex activities were statistically significant (*p* < 0.05).

**Figure 2:**
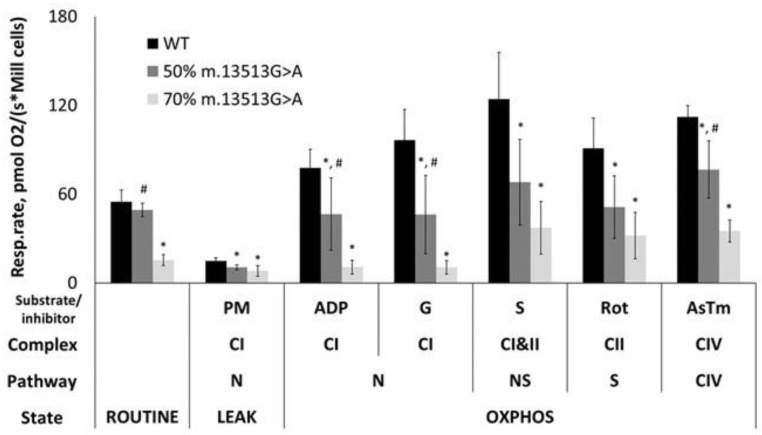
Comparative respiration rates of cybrid cells harboring 50% and 70% of the m.13513G>A variant and of wild-type (WT) cells. Reduced complex I-dependent oxygen consumption is seen in cells harboring the m.13513G>A variant, with more pronounced effect in cells with 70% heteroplasmy. Data are shown as the mean ± standard deviation. * *p* < 0.05 compared with WT cells; # *p* < 0.05 compared with cells having 70% heteroplasmy by two-sample t-tests. Abbreviations: PM, pyruvate + malate; ADP, adenosine diphosphate; G, glutamate; S, succinate; Rot, rotenone; AsTm, ascorbate + TMPD; CI, complex I; CII, complex II; CIV, complex IV; N, NADH; ROUTINE, physiological coupling state; LEAK, non-phosphorylating substrate-dependent state; OXPHOS, oxidative phosphorylation-dependent state.

**Figure 3:**
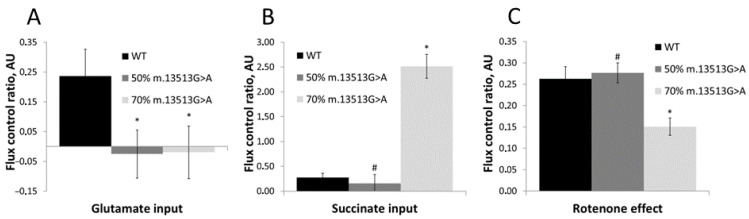
Effect of glutamate (**A**), succinate (**B**), and rotenone (**C**) on respiration rates of cell lines harboring 50% and 70% of the m.13513G>A variant and wild-type (WT) cells. Data are shown as the mean ± standard deviation. * *p* < 0.05 compared with WT cells; # *p* < 0.05 compared with cells having 70% heteroplasmy by two-sample *t*-tests.

Evaluation of mitochondrial respiration in intact cells showed that ROUTINE respiration was 72% lower in cells containing 70% of the m.13513G>A variant than in cells containing WT (*p* < 0.05) (Figure 2). Assessments of mitochondrial functionality in permeabilized cells showed that complex I-dependent oxygen consumption was lower in cell lines with 50% and 70% heteroplasmy. Respiration rates in the OXPHOS state using pyruvate and malate as substrates were 40% and 86% lower in cell lines containing 50% and 70% mutant mtDNA, respectively, than in WT cells (Figure 2). Moreover, respiration rates of both heteroplasmic cell lines did not increase after addition of glutamate (Figure 2 and Figure 3), indicating that the reductions in respiration were directly related to complex I function and not substrate specific metabolism. Further evaluation of the cell line harboring 70% of the m.13513G>A variant showed that rotenone had a significantly reduced effect, whereas succinate had a significantly increased effect on respiration rate (Figure 3). In contrast, the cell line harboring 50% of the m.13513G>A variant showed rotenone and succinate effects similar to the WT cells (Figure 3).

### 3.2. Effect of the Pathogenic mtDNA Variant on Differential Gene Expression

**Figure 4:**
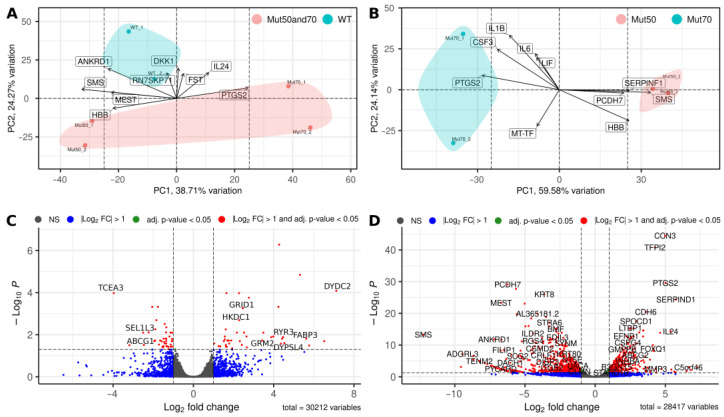
Differential gene expression in cybrid cells harboring 50% and 70% of the m.13513G>A variant and cells containing WT mtDNA. (**A**,**B**) Principal component analysis showing variability between (**A**) heteroplasmic cells containing m.13513G>A variant (50% and 70%) and WT mtDNA and (**B**) cells with 50% heteroplasmy vs. cells with 70% heteroplasmy. (**C**,**D**) Volcano plots for expressed genes (**C**) in the cells with 50% and 70% heteroplasmy vs. cells containing WT mtDNA and (**D**) in the cells with 50% heteroplasmy vs. cells with 70% heteroplasmy.

The effects of the pathogenic mtDNA variants on differential gene expression were assessed by performing transcriptome analyses of cybrid cells containing 50% and 70% of the m.13513G>A variant and cells containing WT mtDNA. Principal component analysis revealed that most variability is explained by the different mutational loads (50% versus 70%) within the heteroplasmic cells (Figure 4A). Furthermore, a second source of variability arises from the difference between WT cells and both cell lines harboring the m.13513G>A variant (Figure 4B).

RNA sequencing analysis of both heteroplasmic cell lines versus WT cells showed that the levels of transcription of 84 nuclear genes were significantly affected (adj. *p* < 0.05) by the m.13513G>A variant (ArrayExpress accession No. E-MTAB-11035). The corresponding volcano plot is shown in Figure 4C. Evaluation of DEGs, defined as those with absolute logFC >2 and FDR < 0.05 cut-offs, showed that the levels of expression of 31 genes were altered more than fourfold, with 25 of those genes being downregulated (log2 FC > 2) and six being upregulated (log2 FC < –2) (Table S2). All of these genes were nuclear.

**Figure 5:**
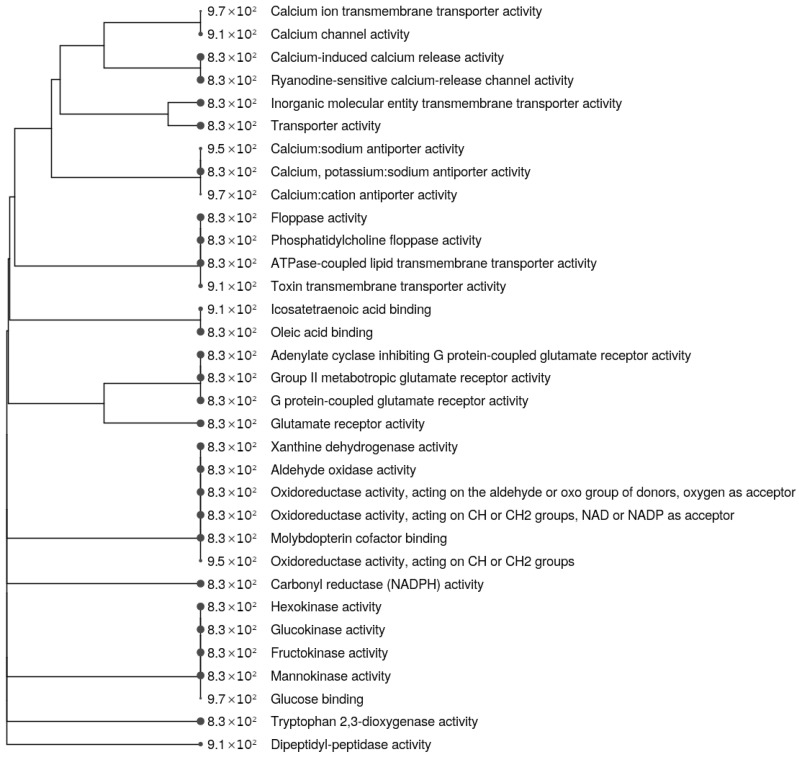
Clustered molecular function Gene Ontology terms of the differentially expressed genes (DEGs) differing significantly in cybrid cell lines harboring 50% and 70% of the m.13513G>A variant in the *MT-ND5* gene compared with WT cells. DEGs were defined as those with absolute logFC > 2 and false discovery rate (FDR) < 0.05; the data were plotted using R package ShinyGO. Top 30 of Molecular Function GO terms are shown (FDR cutoff 0.1). Hierarchical clustering tree is built on similarity of gene sets listed in GO terms.

**Figure 6:**
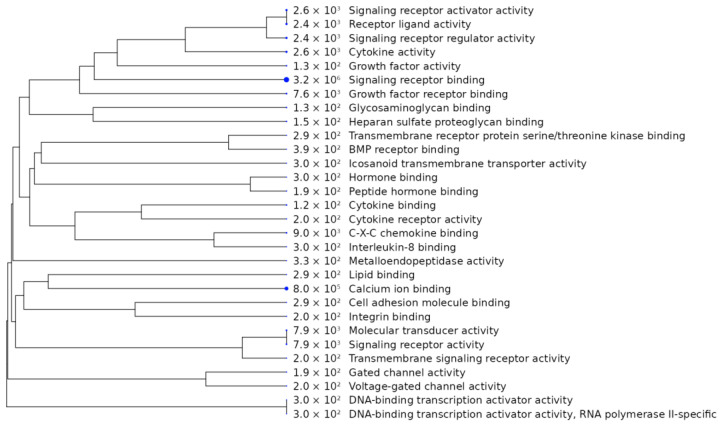
Clustered molecular functions, based on Gene Ontology terms of the differentially expressed genes (DEGs), which differed significantly between cybrid cell lines harboring 50% and 70% of the m.13513G>A variant in *MT-ND5* gene. DEGs were defined as genes with absolute logFC > 2 and false discovery rate (FDR) < 0.05; the data were plotted using R package ShinyGO. Top 30 of Molecular Function GO terms are shown (FDR cutoff 0.1). Hierarchical clustering tree is built on similarity of gene sets listed in GO terms.

To determine the affected molecular pathways and biological functions, the DEGs were subjected to Gene Ontology (GO) enrichment analysis (Figure 5 and Figure 6, Tables S3 and S4). Cells harboring the m.13513G>A variant (both 50% and 70% heteroplasmy) showed enrichment of genes associated with both active and passive transmembrane transporter activity (*GRID1*, *NIPAL4*, *SLC24A4*, *FABP3*, *RYR3*, *ABCG1*), particularly calcium release activity (*RYR3*, *SLC24A4*)(adj. *p* = 0.093). The *HDKC1* gene was also highlighted in the GO enrichment analysis (adj. *p* = 0.093); it encodes kinase, which is involved in carbohydrate (sucrose, fructose, and mannose) metabolism, and is downregulated according to RNAseq results. Cluster analysis of the molecular functions of DEGs, based on GO terms, showed a cluster of genes related to calcium transport/release (Table S3). These findings are in agreement with the GO terms of cellular components, which showed enrichment of integral components of the membrane (Table S3), although it did not reach statistical significance (adj. *p* = 0.430).

The RNAseq analysis of cells harboring 50% versus 70% of the m.13513G>A variant identified 1247 nuclear genes whose transcription levels were significantly affected (adj. *p* < 0.05; Figure 4D). 437 of these genes had levels of expression that were altered more than four-fold (Table S2).

Ten (*DYDC2*, *TCEA3*, *GRID1*, *HKDC1*, *SEL1L3*, *FABP3*, *RYR3*, *GRM2*, *DYPSL4*, and *ABCG1*) of the thirty-one most pronounced DEGs were selected for further evaluation. These genes were chosen because of their putative capacity to be involved in cellular physiological and pathological processes. Genes involved in cancer physiology, however, were excluded, as changes in their expression might be associated with the cancerous nature of ρ0 cells used for the production of cybrid cells. The results of RNA expression analysis of the selected genes were validated using Selfie-digital PCR in freshly propagated cybrid cells (Figure S1). Only four genes yielded results resembling the general trend demonstrated by the RNA seq analysis. Compared with WT cells, the *TCEA3*, *SEL1L3*, and *ABCG1* genes were upregulated, whereas the *FABP3* gene was downregulated, in cells bearing the m.13513G>A variant. The *GRM2* gene was upregulated in the cells harboring 50% of the variant, but downregulated in the cells harboring 70% heteroplasmy of the variant, as well as being downregulated in the RNA seq analysis.

## 4. Discussion

The genetic variant m.13513G>A, first described by Santorelli et al. in a patient with MELAS [49], has been reported to be the most common pathogenic variant in the *MT-ND5* gene [50]. This variant has later been identified in many individuals with heterogeneous clinical manifestations, including Leigh syndrome [51], Wolff Parkinson-White (WPW) syndrome [52], and LHON, as well as overlapping phenotypes [49]. Later, this spectrum was expanded by the addition of nephropathy [53,54,55], sensorineural deafness, and subcortical and cerebellar atrophy, features commonly seen in MELAS [56]. Usually, the heteroplasmic load of the mutant variant shows a good correlation with disease severity and the spectrum of clinical phenotypes, ranging from fatal neonatal forms to asymptomatic cases [57]. Although most pathogenic mtDNA variants must be present at high heteroplasmy levels (>90%) to cause disease, mutated m.13513G>A loads as low as 30–50% can cause complex I deficiency [58]. Moreover, even lower levels of heteroplasmy (~20–30%) have been reported in patients with isolated LHON [59,60,61].

The present study analyzed the effects of the m.13513G>A variant on the OXPHOS system and alterations in cell transcriptional profiles by comparing transmitochondrial cybrid cell lines harboring 50% and 70% of mutated DNA with WT cells. Increased levels of heteroplasmy were found to result in reduced complex I functionality. As expected, complex I activity was reduced in both mutant cell lines, as the *MT-ND5* gene encodes a complex I subunit essential for complex I activity [62]. Furthermore, both mutant cell lines showed reduced complex III activity, and cells with 70% heteroplasmy showed decreases in complex IV and complex I + III activities. Complexes I, III, and IV form respiratory supercomplexes [63]. Although complex I dysfunction does not usually lead to disturbed complex III and complex IV functionality, mutations in nuclear gene *NDUFS4*, which encodes a structural subunit of complex I, were found to cause combined complex I and III deficiencies [64,65,66]. We suggest that the m.13513 G>A mutation in the *MT-ND5* gene may also cause secondary complex III and complex IV deficiencies due to impaired supercomplex formation.

High-resolution respirometry confirmed the complex I defect in both mutant cell lines, with both showing reductions in complex I-dependent oxygen consumption, and cells with 70% heteroplasmy showing little response to rotenone addition. Our obtained data were consistent with previously published results of high-resolution respirometry measurements in fibroblasts harboring the *MT-ND5* m.13513G>A variant, where complex I contribution to respiration was reduced, and the total mitochondrial mass was diminished, leading to a defect in complexes I, III + V, and IV [67]. Furthermore, induced pluripotent stem cells (iPSC) generated from these fibroblasts had reduced basal respiration and a prominent defect in the activity of complexes I and III. Further confirming this, the induced neuron cells generated from these iPSCs also showed complex I deficiency [67].

Moreover, cells with 70% heteroplasmy demonstrated a significantly elevated input of succinate, suggesting a compensatory mechanism of the mitochondria for the production of ATP, as both complex I and complex II are entry points for electrons to the electron transport chain. Complex I deficiencies can be bypassed by supplying the cells with substrates for complex II, such as cell-permeable succinate prodrugs [68]. Interestingly, in contrast to cells with 70% heteroplasmy, cells with 50% heteroplasmy showed sensitivity to rotenone in a similar manner to WT cells, and did not have increased succinate input. This would indicate that, despite diminished complex I-dependent respiration, cells harboring 50% of the m.13513G>A variant are still able to produce enough energy through complex I-dependent oxygen consumption. The considerable difference in effect after succinate input seen in both heteroplasmic cell lines may be explained by the well-known fact that symptoms of mitochondrial diseases, and therefore also the biochemical manifestations, become more severe as the heteroplasmy level grows [17,26,69]. We hypothesize there might be a threshold between 50% and 70% heteroplasmy for the m.13513G>A variant that affects the ability to produce energy through complex I-dependent oxygen consumption and the need for compensation through complex II-dependent respiration. This would be supported by the fact that the affected patient, described by us in detail previously, carries 60% heteroplasmy for the m.13513G>A variant [28].

Little is known about changes in gene expression levels, associated with Leigh syndrome, and in particular with the m.13513G>A variant in the *MT-ND5* gene. Wahedi et al. performed transcriptome analysis of 113 genes, currently associated with Leigh syndrome, and showed neuronal specificity of target genes, with significant enrichment in hippocampal and somatosensory pyramidal neurons as well as interneurons of the brain [70]. A previously described, transcriptomic profile analysis of fibroblasts harboring pathogenic variants in the *LRPPRC* gene, associated with Leigh syndrome, French-Canadian type (MIM #220111), obtained a set of DEGs and identified *NDUFA4L2* gene as a target for further functional studies [71].

The RNA sequencing analysis of cells harboring 50% versus 70% of the m.13513G>A variant identified 1247 nuclear genes whose transcription levels were significantly affected, with 437 of them having expression levels altered more than four-fold.

The patient, who participated in our research as a donor of altered mtDNA, which was used for creation of cybrid cell lines, had a heteroplasmy level of 60% in the blood and exhibited features of Leigh syndrome. Confirming this, our studies on OXPHOS system functionality revealed that cells with both 50% and 70% heteroplasmy levels showed reduced complex I and III activities as well as complex I related defect in oxygen consumption. Additionally, it was previously described that, cells harboring 20–30% of the m.3243A>G variant were found to have mutually similar transcriptional profiles [26]. Furthermore, cells harboring 50–90% of the same variant also had mutually similar transcriptional profiles, although these were different from those in cells harboring 20–30% of this variant. This suggests that gradual increases in mtDNA heteroplasmy levels are associated with phased alterations in nDNA and mtDNA gene expression profiles [26]. Taking into consideration all the above-mentioned arguments, we suggest that more weight is to be given to the results of combined RNA sequencing analysis from cells with 50% and 70% heteroplasmy, compared with WT cells. Transcriptome analysis revealed that, compared with WT cells, the levels of expression of 84 nuclear genes were significantly altered in cells bearing the m.13513G>A variant. GO enrichment analysis and clustering of selected DEGs showed the involvement of genes related to calcium transport and release activity. This was not unexpected, as mitochondrial dysfunction was shown to alter levels of intracellular calcium [72]. Cybrids harboring 50%–90% of the 3243A>G variant showed increased expression of glycolytic genes, indicating bioenergetic adaptation by these cells [26]. In contrast, the expression levels of genes involved in glycolysis were not significantly altered in cells bearing the m.13513G>A variant (data not shown).

Selfie-digital PCR analysis of five genes (*TCEA3*, *SEL1L3*, *ABCG1*, *FABP3*, and *GRM2*) showed a trend in expression consistent with the results of transcriptome analysis. The expression of the *TCEA3* gene, which encodes transcription elongation factor, A3 which releases RNA polymerase II from transcriptional arrest [73], is tissue-specific and highly expressed in skeletal muscle, where it promotes the transcription elongation of muscle specific genes by binding to myogenic regulatory transcription factors (MRFs) MyoD and myogenin. It is a required co-factor for MRF-driven gene expression during myogenesis [74]. In proliferating cells, *TCEA3* is expressed at low levels, however, upon differentiation, *TCEA3* is upregulated [74]. Because exercise intolerance and myopathy are often associated with the m.13513G>A variant, we can speculate that upregulation of *TCEA3* expression in cells harboring the variant may serve as a compensatory mechanism for defects in muscle functions. The *SEL1L3* gene, which encodes protein sel-1 homolog 3, suppressor of lin-12-like protein 3, is induced in response to mitochondrial damage [75]. *SEL1L3* was identified as one of ten common mitochondrial defect (CMD) genes, and was shown to contribute to CMD-mediated regulatory mechanism, resulting in mitochondrial defect and subsequent retrograde signals for transcriptional reprogramming during hepatocellular carcinoma progression [75]. The *ABCG1* gene, which encodes ATP binding cassette subfamily G member 1, plays a prominent role in apolipoprotein mediated cholesterol transport and suppresses β-amyloid (Aβ) accumulation. Dysregulated *Abcg1* expression is associated with intracellular cholesterol accumulation in the brains of the APP/PS1 mouse model [76]. The upregulation of the *ABCG1* gene in cells harboring the m.13513G>A variant could be considered a compensatory mechanism, possibly unspecific, for Aβ induced early mitochondria-associated ER membrane dysregulation. The *FABP3* gene, which encodes fatty acid binding protein 3, is critical for the uptake of alpha-synuclein and its aggregation into intracellular filamentous-shaped inclusion bodies in a mouse cell model, as well as being involved in MPP+-induced neuronal retraction, mitochondrial activity, and reactive oxygen species (ROS) formation [77]. *Fabp3* knockout mice showed decreased alpha-synuclein oligomerization and neuronal degeneration of tyrosine hydroxylase positive neurons in vivo. Published data indicate that FABP3 is critical for preventing synucleinopathies; therefore, its downregulation in cells harboring the m.13513G>A variant could lead to impaired alpha-synuclein uptake and formation of aggregates [77]. This in turn could disturb the normal functioning of neurons and could be associated with neuronal dysfunction observed in Leigh syndrome. The *GRM2* gene, which encodes glutamate metabotropic receptor 2, plays an important role in normal brain function, and is a potential target for the development of drugs for neurological and psychiatric diseases [78]. In a recent study, it was shown that *GRM2* is negatively regulated by miR-342-*p*, one of miRNA expressed in the epileptic hippocampus. Upon pharmacological induction of tonic–clonic seizures in rats, the downregulation of miR-342-*p* and upregulation of its target gene *GRM2* were observed [79]. These results suggested a possible involvement of *GMR2* in epileptogenesis. Seizures are one of the clinical manifestations associated with the m.13513G>A variant. Therefore, we propose that upregulation of *GRM2* may play a role in their development.

In summary, mutant cell lines harboring the m.13513G>A variant of the *MT-ND5* gene showed defects in the OXPHOS system, which had a more pronounced effect in cells bearing 70% than 50% heteroplasmy. These findings are in agreement with results showing that higher levels of heteroplasmy cause more severe symptoms. The pathogenic mtDNA variant had pronounced effects on nuclear gene expression in cybrid cell lines, with DEGs in cells harboring the m.13513G>A mtDNA variant generally reflecting the physiological processes associated with damaged mitochondria.

## Data Availability

Publicly available datasets were analyzed in this study. These data can be found at [ArrayExpress/ E-MTAB-11035].

## Supplementary Materials

The following supporting information can be downloaded at: https://www.mdpi.com/article/10.3390/cimb45030115/s1. Figure S1: The dynamics in transcription level of selected genes evaluated by Selfie-digital PCR in cybrid cells bearing m.13513G>A mtDNA variant. Graph shows representative experiment. Table S1: The primer used for Selfie-digital PCR. Table S2: List of differentially expressed genes. Selected genes that correspond to chosen criteria (padj < 0.05 and absolute logFold change > 2) are shown in red. Table S3: Ontology terms determined for the selected 31 DEGs differing significantly in cybrid cell lines harboring 50% and 70% of the m.13513G>A variant in the MT-ND5 gene compared with WT cells, ranked by statistical significance (*p* < 0.05 and absolute logFold change > 2). Table S4: Gene Ontology terms determined for the selected 437 DEGs that differed significantly between cybrid cell lines harboring 50% and 70% of the m.13513G>A variant in MT-ND5 gene, ranked by statistical significance (*p* < 0.05 and absolute logFold change > 2).
