# Supplementary material for: Impact of the m.13513G>A Variant on the Functions of the OXPHOS System and Cell Retrograde Signaling
Source: Curr Issues Mol Biol. 2023 Feb 22;45(3):1794–809. doi: 10.3390/cimb45030115 (PMC10047405; doi:10.3390/cimb45030115)
Supplement: Supplementary file 1 [file cimb-45-00115-s001.zip › cimb-2149402-Table S1.pdf]

Table S1. The primer used for Selfie-Digital PCR

Gene	Amplicon size (bp)	Forward primer sequence 5'-3'	Reverse primer sequence 5'-3'
GAPDH	74	GATGGCCCCTCCGGGAAACT	GCGCCAGTAGAGGCAGGGAT
PPIA	70	ACCGCCGAGGAAAACCGTGT	CGTCGACGGCAATGTCGAAGAA
RPL30	92	GTGGGAAGTACGTCCTGGGGT	AGCTGGGCAGTTGTTAGCGA
DYDC2	61	GCTTTGGAAATTGCCTGGCCC	GGGTCACTGGGCCGAACCTT
TCEA3	96	AGGAAGAAGAAAGAGGAAGCGCTGA	ACCATCCCACTCAGACAGAGTTCA
GRID1	91	CTCTCGGCCCTGGAGATGGG	AGGTGCTGACCGAGAGCTGG
HKDC1	100	TGGGGGAGATTGTGCGGCAG	TCGAAGATGCCCCTGGTCCG
SEL1L3	87	GCTGTTCTGGGGGCAGCAAG	GGATCCTCCGTCTCCAGGGC
DHRS2	85	GGGCTGGTTTCATCCCTGTGC	TACCCGGTTAGCCAGGACGC
FABP3	74	CCTTGCACGCCTGCTCTCTT	GTGCCCAGGAAAGCGTCCAC
RYR3	65	CGACCAGGTGGACCCCTTCC	AAGAAGAGGCCCAGCCCACC
GRM2	82	GGCCATGGGATCGCTGCTTG	CAGCACCTTCTTGGCTGGGC
DPYSL4	74	TGGTCCTTCCTGGTGGCGTT	GTCGTCAGCCGGTGTCATGC
ABCG1	80	ATGACGGAGCCCAAGTCGGT	AGCAGGTCCGTCTCAGTGGC
