# Supplementary material for: Impact of the m.13513G>A Variant on the Functions of the OXPHOS System and Cell Retrograde Signaling
Source: Curr Issues Mol Biol. 2023 Feb 22;45(3):1794–809. doi: 10.3390/cimb45030115 (PMC10047405; doi:10.3390/cimb45030115)
Supplement: Supplementary file 1 [file cimb-45-00115-s001.zip › cimb-2149402-title.pdf]

Supplementary Table S1. The primer used for Selfie-digital PCR.

Supplementary Table S2. List of differentially expressed genes (DEGs). Selected genes that correspond to chosen criteria (padj<0.05 and absolute logFold change >2) are shown in green. Negative log2FoldChange are shown in red.

Supplementary Table S3. Gene Ontology terms determined for the selected 31 DEGs differing significantly in cybrid cell lines harboring 50% and 70% of the m.13513G>A variant in the MT-ND5 gene compared with WT cells, ranked by statistical significance (p<0.05 and absolute logFold change >2).

Supplementary Table S4. Gene Ontology terms determined for the selected 437 DEGs that differed significantly between cybrid cell lines harboring 50% and 70% of the m.13513G>A variant in MT-ND5 gene, ranked by statistical significance (p<0.05 and absolute logFold change >2).

Figure S1. The dynamics in transcription level of selected genes evaluated by Selfie-digital PCR in cybrid cells bearing m.13513G>A mtDNA variant. Graph shows representative experiment.
