# Supplementary figures and images for: Impact of the m.13513G>A Variant on the Functions of the OXPHOS System and Cell Retrograde Signaling
Source: Curr Issues Mol Biol. 2023 Feb 22;45(3):1794–809. doi: 10.3390/cimb45030115 (PMC10047405; doi:10.3390/cimb45030115)

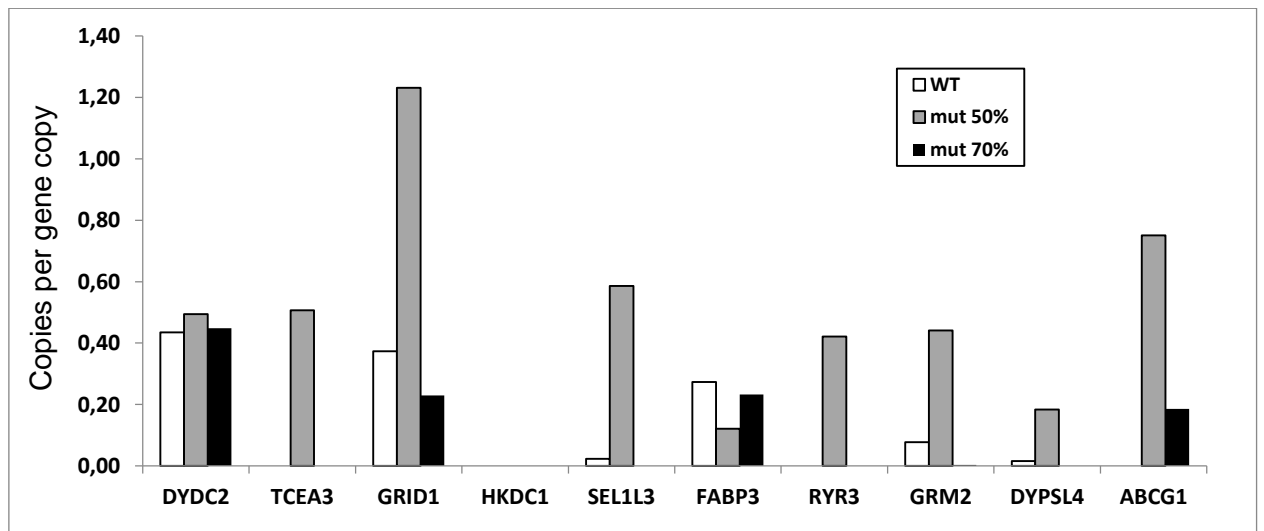

Supplement: Supplementary file 1 [file cimb-45-00115-s001.zip › Kidere et al_2022_Figure S1.pdf]
